# Preparation and Performance of Biomimetic Zebra-Striped Wood-Based Photothermal Evaporative Materials

**DOI:** 10.3390/biomimetics10050334

**Published:** 2025-05-20

**Authors:** Zebin Zhao, Wenxuan Wang, Zhichen Ba, Yuze Zhang, Hongbo Xu, Daxin Liang

**Affiliations:** 1 Key Laboratory of Bio-Based Material Science and Technology (Ministry of Education), Northeast Forestry University, Harbin 150040, China; zhaozebin100@126.com (Z.Z.); wxwang@nefu.edu.cn (W.W.); bazc_@nefu.edu.cn (Z.B.); zyz050611@163.com (Y.Z.); 2 School of Chemistry and Chemical Engineering, Harbin Institute of Technology, Harbin 150001, China; iamxhb@hit.edu.cn

**Keywords:** wood, biomimicry, solar interface evaporation

## Abstract

An efficient solar water evaporator is an important strategy for addressing the problem of water shortage. Constructing high-performance solar interfacial evaporators through bionic design has become a crucial approach for performance enhancement. Through the study of zebra patterns, it has been found that the black-and-white alternating patterns generate vortices on the surface of the zebra’s skin, thereby reducing the temperature. By utilizing the vortices brought about by the temperature difference, the design of a solar water evaporator is created based on the bionic zebra pattern, so as to improve its water evaporation performance. In this work, green and sustainable wood is used as the base of the evaporator, and the bionic design of zebra stripes is adopted. Meanwhile, the following research is conducted: The wood is cut into thin slices with dimensions of 30 × 30 × 5 mm^3^, and a delignification treatment is performed. Tannic acid-Fe ions are used as the photothermal material for functionalization. A series of stable patterned water evaporators based on delignification wood loaded with tannic acid-Fe ion complex (TA-Fe^3+^) are successfully prepared. Among them, the wood-based solar water evaporator with 3 mm zebra stripes exhibits excellent photothermal water evaporation performance, achieving a water evaporation rate of 1.44 kg·m^−2^·h^−1^ under the illumination intensity of one sun. Its water evaporation performance is significantly superior to that of other coating patterns, proving that the bionic design of zebra patterns is effective and can improve water evaporation efficiency. This work provides new insights into the development of safe and environmentally friendly solar interfacial water evaporation materials through bionic design.

## 1. Introduction

Although around 71% of the Earth’s surface is covered by water, only about 3% of this total amount is fresh water that can be directly used by humans, and freshwater resources are always in short supply. What is worse, with the rapid increase in the demand for water in household and industrial sectors, coupled with the continuous severity of water pollution, the depletion of freshwater resources has become one of the most urgent global challenges [1,2]. In China, due to differences in population density and the distribution of water resources, the problem of freshwater resource shortage is particularly serious, and the per capita available freshwater resources are only a quarter of the global average [3]. This obvious gap exacerbates the challenges of water resource management and sustainable utilization, and expanding the sources of environmentally friendly freshwater resources has become an important research direction [4].

In recent years, solar-driven interfacial photothermal evaporation technology has gained significant attention in the fields of seawater desalination and wastewater treatment due to its sustainability, environmental friendliness, and low cost [5]. A typical solar interfacial evaporation system consists of a photothermal layer and a water transport layer [6]. The photothermal materials used in the photothermal layer primarily include plasmonic metal-based materials [7], carbon-based photothermal materials [8], semiconductor-based photothermal materials [9], and organic photothermal materials [10]. Cellulose gels, wood, and similar materials are commonly selected as the substrate materials for the water transport layer due to their advantageous properties, including hydrophilicity, thermal insulation, and porosity [11,12]. Lu [13] utilized naturally sourced wood that underwent a carbonization process as a solar absorber. Under a solar irradiance of 1 sun, the carbonized wood exhibited a water evaporation rate of 1.03 kg m^−2^ h^−1^. Gao [14] synthesized a wood-based photothermal evaporator by loading Fe^3+^-catechol complexes onto wood. Under one solar intensity, the evaporator demonstrated a water evaporation rate of 1.25 kg m^−2^ h^−1^. Tannic acid (TA), a naturally occurring plant-derived polyphenol, is widely sourced from various botanical materials and is recognized for its antioxidant properties, adhesive capability, and biocompatibility, making it utilized extensively in biomedical and food-related applications [15]. Owing to the presence of multiple phenolic hydroxyl groups, TA exhibits a strong chelating ability with diverse metal ions such as Fe^3+^, enabling the formation of metal–polyphenol redox complexes [16]. These complexes can serve as effective initiators for free-radical polymerization, significantly enhancing reaction rates. The chelate formed between TA and Fe(III), commonly referred to as complex of tannic acid and iron ions (TA–Fe^3+^), has demonstrated promising photothermal conversion efficiency, suggesting its applicability in areas such as photothermal therapy, antimicrobial treatment, and solar-driven water evaporation [17].

The exploration of strategies to enhance material performance has led to the development of various biomimetic approaches, which involve identifying solutions in nature and extracting these concepts to address human challenges [18]. Wang [19] developed an integrated, highly efficient, self-floating, jellyfish-inspired solar absorber based on partially carbonized Enteromorpha aerogel, which transforms waste biomass into photothermal materials with excellent performance. In recent years, research has focused on the temperature regulation function of zebra skin stripes. A study conducted in Kenya on live zebras revealed a temperature differential between the stripes, primarily caused by air flow-induced turbulence in the zebra’s fur, which enhanced the cooling effect through evaporation [20]. Additionally, a study applied biomimetic zebra stripe designs to building materials in Panama. This design reduced the exterior surface temperature of buildings by approximately 5 °C, while also improving energy efficiency by decreasing cooling energy consumption [21].

These studies highlight the potential of zebra stripes to utilize the air flow effects generated by temperature differences to accelerate evaporation and regulate temperature. In this paper, TA-Fe^3+^ with high biocompatibility is used as the photothermal material, and delignified wood that can self-float and is environmentally friendly is used as the substrate. The two are combined through a simple coating method to form a stable composite photothermal material. Moreover, by controlling the coating method, the bionic construction of zebra stripes is precisely carried out on the surface of the wood. Through the photothermal reaction, it is found that introducing the bionic design of zebra stripes into the solar evaporator can greatly increase its water evaporation efficiency, and it is stable and reliable. This study shows that the bionic construction of zebra stripe structures can improve the light utilization efficiency of solar evaporators, verifies the photothermal performance of samples with different surface textures, examines the performance of the bionic zebra stripe solar interfacial evaporation system, and provides new ideas for the design of solar water evaporators.

## 2. Materials and Methods

### 2.1. Materials

The Balsa wood used in this experiment is sourced from Ecuador, South America. Sodium acetate (CH_3_COONa), sodium chlorite (NaClO_2_), sodium alginate (SA), sodium bicarbonate (NaHCO_3_), and ferric chloride hexahydrate (FeCl_3_·6H_2_O) were purchased from Aladdin (Shanghai, China). Acetic acid (CH_3_COOH) was purchased from Fuyu (Tianjin, China) and tannic acid (TA) was acquired from Macklin (Shanghai, China). All reagents used were of analytical grade.

### 2.2. Preparation of Balsa-TA-Fe^3+^

Preparation of wood substrate: Using the hypochlorite environment to conduct delignification treatment on the wooden substrate is a commonly used approach; refer to the method proposed by Li et al. [22]. The Balsa wood was cut into 30 × 30 × 5 mm^3^ cross-sectional pieces and placed in a solution containing 1.5 wt% NaClO_2_, 0.3 wt% CH_3_COOH, and 0.68 wt% C_2_H_3_NaO_2_ in 800 mL of water. The mixture was then heated in a water bath at 70 °C for 4 h to remove lignin. This process was repeated three times. Afterwards, the treated wood was removed and washed with distilled water to obtain a pure white wood substrate. The substrate was then placed on a non-woven fabric and dried in a freeze-dryer at −50 °C.

Preparation of TA-Fe^3+^: 10 mL of 0.1 mol L^−1^ tannic acid solution was mixed with 20 mL of 1 mol L^−1^ FeCl_3_ solution. The pH of the mixture was adjusted to 7 using a 0.65 mol L^−1^ NaHCO_3_ solution. The reaction products were separated by precipitation, and the resulting precipitate was washed four times with deionized water, followed by centrifugation. Finally, the precipitate was dried in a freeze-dryer at −50 °C.

Preparation of TA-Fe^3+^ with different patterns: The prepared TA-Fe^3+^ was added to a 2%wt SA solution and stirred until thoroughly dispersed. The resulting mixture was then coated onto the surface of the Balsa wood. The coating was applied in four different patterns: along the central axis with a half-coating, along the diagonal axis with a half-coating, zebra stripes with a width of 5 mm, and zebra stripes with a width of 3 mm.

### 2.3. Characterization

The surface morphology of the sample was investigated using a scanning electron microscope (Zeiss Supra 55, Zeiss, Oberkohen, Germany). The functional group composition of the compounds was recorded using a Fourier-transform infrared spectrometer (Spectrum 100, PerkinElmer, Waltham, MA, USA). The contact angle was measured using a contact angle goniometer (DSA 100, Kruss, Shanghai, China). Elemental composition analysis of the different materials was performed using X-ray photoelectron spectroscopy (Thermon Kalpa, Thermon, TX, USA). The reflectance (R) and transmittance (T) spectra of the materials were recorded using a UV-3600 spectrophotometer (UV-3600, Shimadzu, Tokyo, Japan), and the absorption efficiency (A) was calculated using the formula A = 1 − R − T. Surface temperature and infrared images were obtained using a thermal infrared imager (FOTRIC 850, Fotric, Shanghai, China).

### 2.4. Solar Evaporation Performance

The experiment was conducted using a xenon lamp light source to irradiate sets of samples under three different light intensities: 0.5 kW m^−2^, 1 kW m^−2^, and 1.5 kW m^−2^. Each sample was irradiated for 300 s, and infrared thermography was used to record the photothermal data. The coated wood-based materials were placed in molds containing 40 mL of distilled water, ensuring the materials floated on the water surface.

Each time, the optical power meter was used to precisely adjust the light intensity before irradiation. Simultaneously, a digital balance was employed to record the mass loss due to water evaporation over a 60 min period under the respective light intensities (0.5 kW m^−2^, 1 kW m^−2^, and 1.5 kW m^−2^), taking the data from the last 30 min after stabilization and recording it. This setup allowed for the measurement of water evaporation rates and the assessment of the photothermal material’s performance under different solar irradiance conditions. The mass loss of water served as an indicator of the steam generation rate, which was used to evaluate the efficiency of the photothermal materials at varying light intensities. Finally, the samples were subjected to cyclic testing.

## 3. Results

### 3.1. Characterization of TA-Fe^3+^-Balsa

#### 3.1.1. SEM of TA-Fe^3+^-Balsa

**Figure 1:**
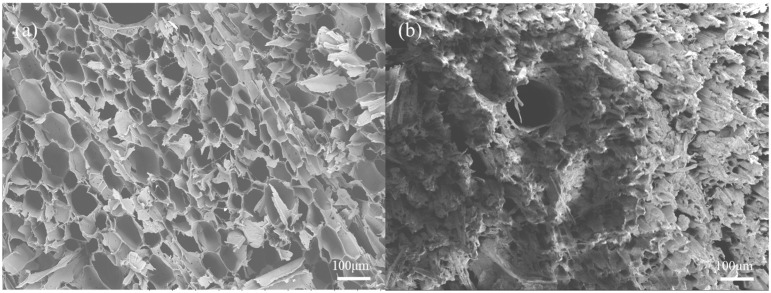
(**a**) SEM of Balsa; (**b**) SEM of Balsa-TA-Fe^3+^.

In order to gain a deeper understanding of the microstructure of the material, a scanning electron microscope (SEM) was used to capture detailed images of the surface structure and morphology of the sample, providing key data for the study of the microscopic characteristics of the material. During the natural growth process, trees form vertically aligned microchannels for transporting water and nutrients from the soil, endowing the wood with a unique anisotropic and porous structure. The balsa wood after delignification treatment is shown in Figure 1a. This figure shows that even after the delignification treatment, the sample retains its original vertically aligned and clearly distinguishable internal pores, and the pipeline structure in the wood for transporting water is not damaged, maintaining a certain strength, which provides an excellent structural basis for water transportation during the evaporation process. As shown in Figure 1b, the TA-Fe^3+^ complex was subsequently introduced onto the surface of the delignified balsa wood to form a double-layer structure, in which the TA-Fe^3+^ complex serves as the photothermal layer and the balsa wood serves as the thermal insulation layer. This configuration is a classic design of the wood-based solar interfacial evaporator. This design can produce effective effects and is currently an efficient method for constructing photothermal water evaporators. SEM has proven the suitability of this method [23].

#### 3.1.2. FTIR of TA-Fe^3+^-Balsa

**Figure 2:**
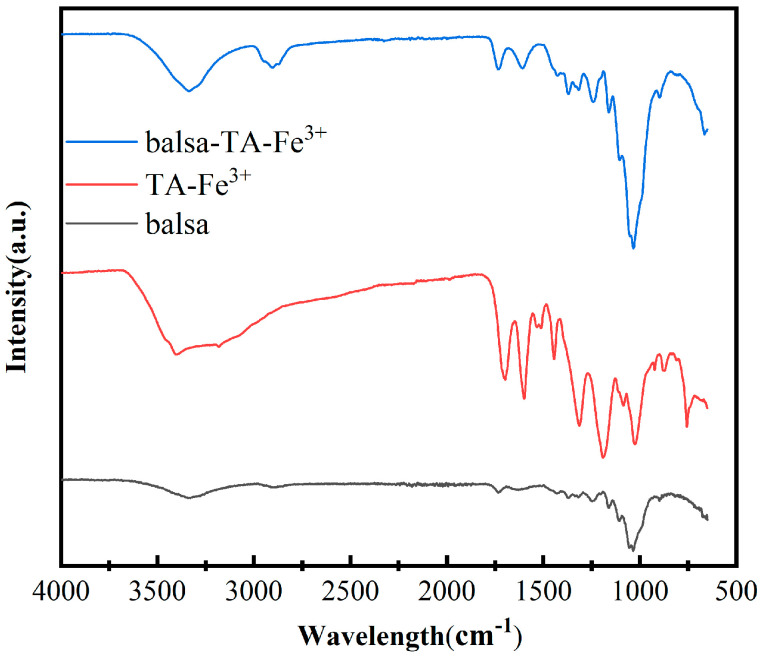
FTIR of balsa, TA-Fe^3+^, and Balsa-TA-Fe^3+^.

The chemical compositions of different samples were analyzed using Fourier transform infrared spectroscopy (FT-IR). In the experiment, the samples were placed on a sample holder equipped with a diamond accessory. The pressure sensor was adjusted to an appropriate height. The specific test parameters are as follows: the wavelength range was set from 4000 to 400 cm^−1^ to cover the infrared absorption peaks of most common chemical bonds; the scanning rate was set at 16 scans per minute to ensure the accuracy and reproducibility of the data, and the infrared spectral information of the samples was accurately collected. The results are shown in Figure 2. The balsa wood sample exhibited a broad absorption band near 3400 cm^−1^, corresponding to the stretching vibration of the inherent hydroxyl (–OH) groups in the wood, indicating its hydrophilicity. The TA-Fe^3+^ complex showed a series of distinct absorption peaks in the range of 1500–1200 cm^−1^, which can be attributed to the C=C skeleton vibration of the aromatic ring and the bending vibration of the phenolic hydroxyl (–OH) groups. In addition, the characteristic peaks in the region of 600–800 cm^−1^ were related to the stretching vibration of the Fe–O bond, confirming the coordination interaction between tannic acid and Fe^3+^ ions.

In the spectrum of the balsa wood-TA-Fe^3+^ composite, the presence of the –OH absorption band of the wood and multiple characteristic peaks of the TA-Fe^3+^ complex indicates that the photothermal agent has been effectively integrated into the wood matrix, suggesting that TA-Fe^3+^ is compounded with balsa wood through hydrogen bonding. It is worth noting that the enhanced absorption in the region of 1200–800 cm^−1^ further supports the enrichment of functional groups in the composite, indicating the success of the chemical modification. These findings provide a solid structural basis for subsequent research on the photothermal properties and interfacial evaporation behavior.

#### 3.1.3. XPS of TA-Fe^3+^-Balsa

**Figure 3:**
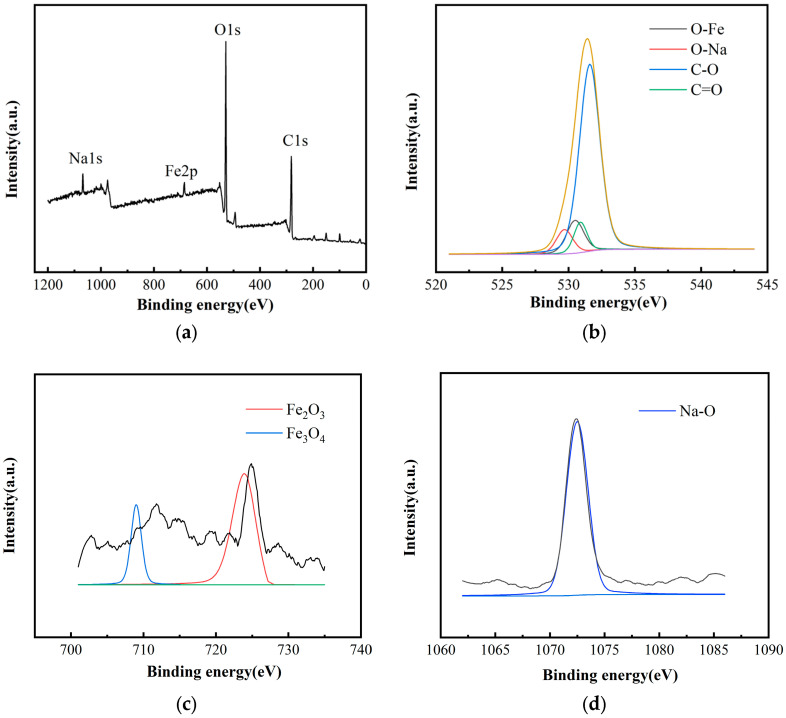
XPS of Balsa-TA-Fe^3+^, (**a**) survey XPS of TA-Fe^3+^-Balsa, (**b**) high resolution spectrum of O 1S, (**c**) high resolution spectrum of Fe, (**d**) high resolution spectrum of Na.

The elemental composition and valence state information of the samples were accurately determined using X-ray photoelectron spectroscopy (XPS). During the testing process, the XPS instrument irradiates the sample surface to generate photoelectrons, which are then analyzed based on their kinetic energy and quantity as they escape from the surface. These data provide detailed information about the sample’s surface chemical composition, the valence states of elements, and surface structure. By analyzing the obtained spectra, the presence and chemical state of each element can be identified, providing important insights for further studies on the surface characteristics, reactivity, and performance of the material in specific applications. As shown in Figure 3, the O 1s peak typically reveals the oxygen-containing functional groups on the surface of the sample. When the tannic acid-iron (TA-Fe^3+^) complex is loaded onto the wood, the O 1s peak may show new changes associated with oxidation or coordination, with peaks corresponding to iron oxides appearing at 532 eV, and a Na-O peak appearing near 529 eV. The Fe 2p peak shows the oxidation state of iron at 711 eV. In the high-resolution Fe 2p spectrum, characteristic peaks observed at approximately 711.2 eV and 724.4 eV correspond to Fe_3_O_4_ and Fe_2_O_3_, respectively, indicating the presence of Fe^3+^ along with a small amount of Fe^2+^ in the material. This confirms the formation of a stable coordination complex between tannic acid and Fe^3+^. Additionally, a prominent peak near 1071 eV in the high-resolution Na 1s spectrum can be attributed to O–Na bonding, which is presumed to originate from sodium alginate being present in the coating material. These results collectively indicate the successful loading of the TA-Fe^3+^ complex onto the wood surface after mixing with SA. The results are consistent with the literature [24].

#### 3.1.4. Hydrophilicity Test of TA-Fe^3+^-Balsa

**Figure 4:**
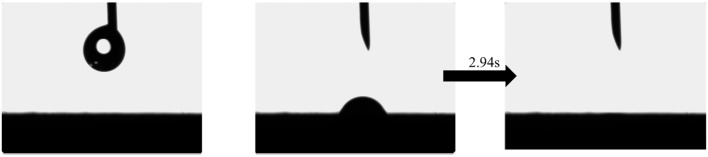
Hydrophilicity test of TA-Fe^3+^-Balsa.

The hydrophilicity of the samples was evaluated using a DSA 100 contact angle measuring instrument, and the corresponding test images were recorded. During the test, water droplets were evenly dropped onto the surface of the samples. The instrument captured the shape of the droplets through a high-precision imaging system and measured the time required for complete absorption. As shown in Figure 4, the bass-TA-Fe^3+^ composite has a high water absorption rate, indicating that the surface of the sample has a sufficiently strong hydrophilicity. Another important advantage is that, as shown in the figure, the wood can absorb a water droplet in only 2.94 s, which indicates that the sample has a good water transportation capacity. This can provide sufficient water for interfacial evaporation and also create space for the escape of water vapor, enabling the enhancement of the water evaporation efficiency.

### 3.2. Analysis of Photothermal Performance

#### 3.2.1. Heating Performance

**Figure 5:**
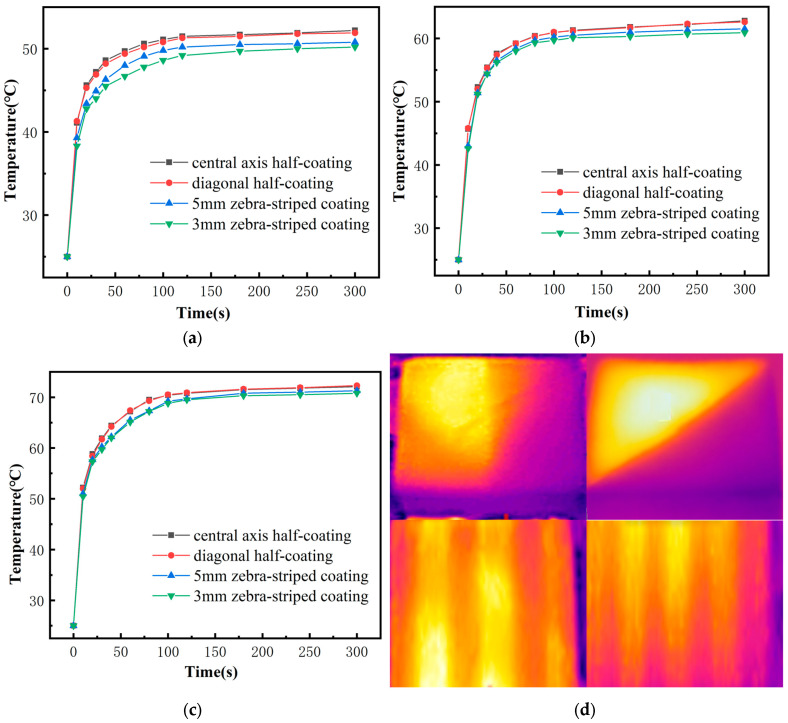
(**a**) Temperature rise curves under 0.5 kW m^−2^ light intensities; (**b**) Temperature rise curves under 1 kW m^−2^ light intensities; (**c**) Temperature rise curves under 1.5 kW m^−2^ light intensities; (**d**) Infrared thermal imaging of the samples.

As shown in Figure 5, all samples can rapidly increase in temperature within a short period. Under one sun intensity, the zebra-pattern-coated samples can quickly heat up to around 60 °C in just tens of seconds, slightly lower than the half-coated samples. The light intensity has a significant impact on the photothermal performance of the samples. The higher the light intensity, the faster the temperature increases, and the higher the final temperature. By comparing the photothermal data at three different light intensities, it can be observed that the higher the light intensity, the faster and more significant the temperature increase on the sample surface. With the increase in light intensity, the differences between the different samples become smaller. The samples with the half-coated central axis and half-coated diagonal axis show similar temperature rise trends. The zebra-pattern-coated samples have slightly slower temperature rise rates and lower final temperatures compared to the two half-coated samples at all light intensities. Furthermore, the denser the zebra pattern, the slower the temperature rise rate and the lower the final temperature. The figure shows the thermographic images of samples with different surface morphologies. This is consistent with the conclusion from Horvath [25] on the preliminary exploration of zebra stripes, which suggests that under light exposure, black-and-white fur has a slightly lower temperature than pure black fur, with a small but not significant difference between them.

#### 3.2.2. UV-Vis-NIR of TA-Fe^3+^-Balsa and Cycling Stability

**Figure 6:**
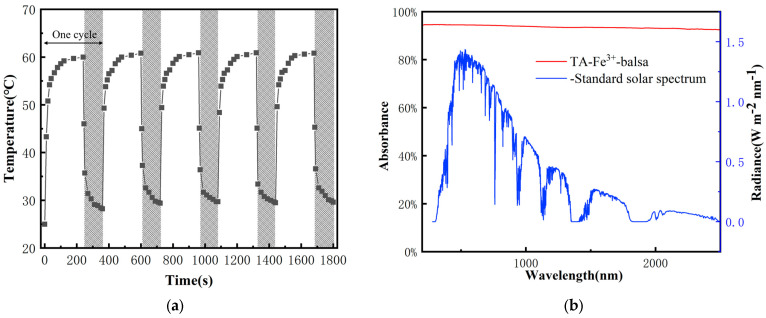
(**a**) Heating/cooling cycle temperature profile; (**b**) AM1.5G standard solar spectrum and UV-Vis-NIR absorption spectrum of Balsa-TA-Fe^3+^.

For the water evaporator, reliable performance can bring about stable water evaporation efficiency. Therefore, a cyclic performance test was carried out on the sample with a 3 mm zebra stripe coating, and the results are shown in Figure 6a. Through repeated heating/cooling cycles (240 s of illumination followed by 120 s of darkness), the sample exhibited stable photothermal conversion ability and excellent recyclability during 5 cycles, indicating that the water evaporator is stable under long-term operation.

To further explore the light absorption characteristics of Balsa-TA-Fe^3+^, an ultraviolet-visible-near-infrared (UV-Vis-NIR) spectroscopy was conducted (Figure 6b). The spectral range (200~2500 nm) covers the main bands of solar radiation energy distribution. The samples exhibit high absorption rates across the entire spectrum. They demonstrate good absorption in the near-infrared (NIR) region (800~2500 nm), although the absorption rate slightly decreases in the long-wavelength NIR region (1085~2500 nm) to 90~93%. However, solar energy distribution in this region is relatively low. In the region where solar radiation energy is most concentrated (400~800 nm), the samples achieve an absorption rate of up to 95%, indicating exceptionally strong solar light absorption capability. This enables efficient harvesting of energy from sunlight and its conversion into thermal energy.

### 3.3. Interfacial Evaporation Performance

**Figure 7:**
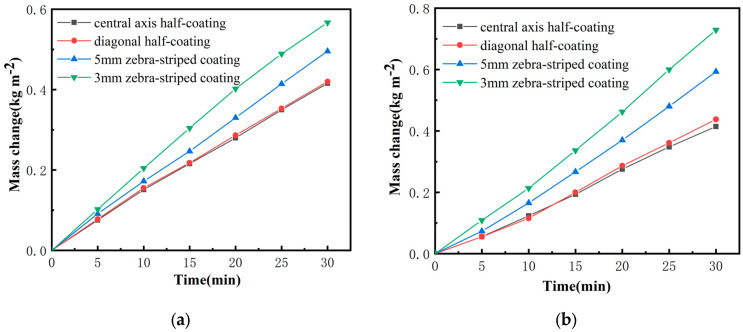

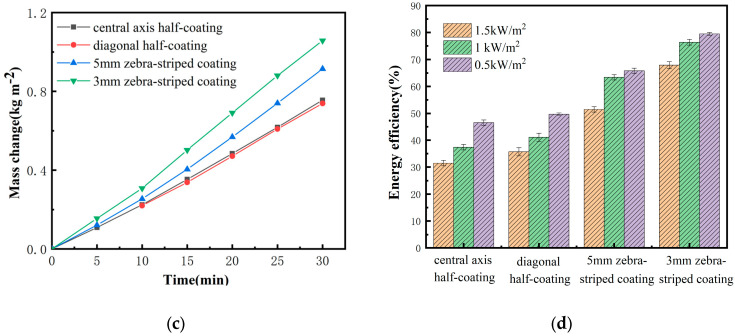
(**a**) Evaporation capacity under 0.5 kW m^−2^ light intensities; (**b**) Evaporation capacity under 1 kW m^−2^ light intensities; (**c**) Evaporation capacity under 1.5 kW m^−2^ light intensities; (**d**) Photothermal efficiency.

Figure 7a–c shows the variation in water evaporation over time for TA-Fe^3+^-Balsa samples with different surface morphologies under various light intensities from a xenon lamp. As the light intensity increases, the evaporation rate of all samples also increases.

Under all three light intensities, the samples exhibit similar trends. The evaporation rate of the 3 mm zebra-stripe sample is higher than that of the 5 mm zebra-stripe sample, which in turn is higher than that of the half-coated sample. The water evaporation rate of the 3 mm zebra-stripe coated sample under 1 kW/m^2^ light intensity is 1.44 kg m^2^ h^−1^, while the 5 mm zebra-stripe coated sample has a rate of 1.18 kg m^2^ h^−1^. The diagonal half-coated sample has a rate of 0.87 kg m^2^ h^−1^, and the central axis half-coated sample has a rate of 0.81 kg m^2^ h^−1^. There is no significant difference in the water evaporation rates between the central axis half-coated and diagonal half-coated samples. However, the zebra-stripe coated samples show a noticeable increase in water evaporation, with the 3 mm zebra-stripe coating exhibiting a greater improvement than the 5 mm zebra-stripe coating. This trend becomes more pronounced with increasing light intensity.

As shown in Figure 7d, although the evaporation rate rises with the increase in the light intensity, the evaporation efficiency gradually decreases as the light intensity increases. This indicates that the thermal energy converted by the photothermal material does not increase linearly with the light intensity. The reason is that a large amount of heat generated by the increase in the light intensity cannot be effectively used for water evaporation, resulting in greater heat loss [26]. However, the four samples still show similar trends under different light intensities, indicating that the gain effect brought by the zebra stripes is reliable. Under the illumination of one sun, the evaporation efficiency of the semi-coated sample is about 41%, while that of the sample coated with 3 mm zebra stripes is increased to 76%. This demonstrates an excellent energy conversion performance. Obviously, the bionic zebra stripe coating method makes a significant contribution to the performance improvement of the solar interfacial evaporator.

As Cobb [20] mentioned in his study on how zebras regulate body temperature, under sunlight, the black-and-white stripes maintain a temperature difference throughout the day, with the greatest contrast occurring during the hottest part of the day. The coexistence of different temperatures leads to air convection on the fur surface, with warm air rising from the hotter stripes (black) and cool air descending onto the cooler stripes (white). The airflow above and between the black-and-white stripes may indeed generate turbulent air vortices due to convection.

These vortices are not cool breezes themselves, but rather serve as reliable media for the exchange of air and water vapor under sunlight, helping to disperse the air saturated with sweat. Therefore, as long as there is sunlight, the function of the stripes is likely to trigger this continuous small-scale air convection. Zebras regulate their body temperature by accelerating the evaporation of sweat. By incorporating the zebra stripe pattern design into the solar evaporation process, this effect can be utilized to enhance performance. Thus, through the bionic coating of zebra stripes, the water evaporation efficiency of the water evaporator can be improved. Even when only half of the wood surface is coated, its performance is still significantly better than that of other materials, as shown in Table 1.

## 4. Conclusions

In this study, the principle of bionic zebra stripes was innovatively introduced into the design and preparation of wood-based photothermal water evaporation materials. By leveraging the temperature difference generated by the zebra stripes, the evaporation efficiency of the water evaporator was increased. During the experiment, the conditions were strictly controlled, and comparative tests were conducted whilst ensuring that the coating area and the amount of photothermal material used were completely the same. The results demonstrated that the samples with zebra stripe structures exhibited extremely significant performance improvements. Both the water evaporation rate and efficiency were substantially enhanced, reaching 1.44 kg m^2^ h^−1^, which was higher than that of samples with other patterns. This indicates the effectiveness of the thermal turbulence induced by the bionic zebra stripe materials. This achievement has opened up a brand-new path for the development of safe, environmentally friendly, and highly efficient solar interfacial water evaporation materials. Meanwhile, this bionic design strategy not only aligns with the concept of green and sustainable development, but also, due to its unique structure, it can be easily integrated with other water treatment methods. It is expected to provide crucial technical support for addressing global water resource issues.

## Figures and Tables

**Table 1:** Evaporation properties of partially composite wood materials.

Types of Wood Composites	Modification Process	Evaporation Rate (kg m^2^ h^−1^)	Ref.
CS-wood	Candle soot-infused wood	0.95	[27]
PPy-wood	In situ composite of PPy	1.01	[28]
AIP-wood	AIP loading	1.42	[29]
rGO-wood	Coating with rGO	1.35	[30]
Balsa-TA-Fe^3+^	Delignified wood loaded with TA-Fe^3+^	1.44	This work

## Data Availability

The data presented in this study are available in the manuscript.
